# The C/C Genotype of the C-1019G (rs6295) Polymorphism of the 5-HT1A Receptor Gene Is Associated with Lower Susceptibility to Depressive Symptoms in a Rural Population in Mexico

**DOI:** 10.3390/neurolint17060087

**Published:** 2025-05-31

**Authors:** Margarita Hernandez-Mixteco, Olga Lidia Valenzuela, Cecilia Luz Balderas-Vazquez, Paola Castillo-Juárez, Sandra Rivera-Gutiérrez, Rocío Liliana García-Reyes, Gilberto Cornejo-Estudillo, Ricardo Jiovanni Soria-Herrera, Moises León-Juárez, Addy Cecilia Helguera-Repetto, Daniel Valencia-Trujillo, Victoria Campos-Peña, Eliud Alfredo Garcia-Montalvo, Jorge Francisco Cerna-Cortés

**Affiliations:** 1 Departamento de Microbiología, Escuela Nacional de Ciencias Biológicas, Instituto Politécnico Nacional, Ciudad de México 11340, Mexico; pcastillo_1307@hotmail.com (P.C.-J.); srivera@ipn.mx (S.R.-G.); rlgarciar@ipn.mx (R.L.G.-R.); vatd921205@hotmail.com (D.V.-T.); 2 Facultad de Ciencias Químicas, Universidad Veracruzana, Orizaba, Veracruz 94340, Mexico; ovalenzuela@uv.mx (O.L.V.); elagarcia@uv.mx (E.A.G.-M.); 3 Coordinación de Ingeniería Biomédica, Universidad Anáhuac, Campus Córdoba, Veracruz 94500, Mexico; cecilia.balderas.v@gmail.com; 4 Escuela José Ma. Pastrana. Secretaría de Educación Pública, Yecapixtla, Morelos 62829, Mexico; gcornejo2381@gmail.com; 5 Facultad de Químico Farmacobiología, Universidad Michoacana de San Nicolas de Hidalgo, Morelia Michoacán 58240, Mexico; ricardo.soria@umich.mx; 6 Departamento de Inmunobioquimica, Instituto Nacional de Perinatología Isidro Espinosa de los Reyes, Ciudad de México 11000, Mexico; moisesleoninper@gmail.com (M.L.-J.); addy.helguera@inper.gob.mx (A.C.H.-R.); 7 Servicio de Microbiología Clínica, Instituto Nacional de Enfermedades Respiratorias, Ciudad de México 14080, Mexico; 8 Escuela Militar de Medicina, Centro Militar de Ciencias de la Salud, Secretaría de la Defensa Nacional, Ciudad de México 11200, Mexico; 9 Laboratorio Experimental de Enfermedades Neurodegenerativas, Instituto Nacional de Neurología y Neurocirugía, Manuel Velasco Suárez, Ciudad de México 14269, Mexico; neurovcp@ymail.com

**Keywords:** depression symptoms, 5-HT1A receptor gene, C-1019G polymorphism, Mexican population

## Abstract

Background: Depression is one of the most prevalent mental health disorders worldwide, affecting a significant proportion of the global population. Its etiology is complex and influenced by the interaction of environmental factors and genetic variations. In Mexico, it has been reported that 41.3% of the population exhibits depressive symptoms. Previous studies have suggested that susceptibility to depression may be associated with the C-1019G (rs6295) polymorphism in the serotonin 1A (5-HT1A) receptor gene. Objective: In this study, we aimed to evaluate the association between the C-1019G polymorphism and depressive symptoms in a rural Mexican population. Methods: Using polymerase chain reaction–restriction fragment length polymorphism (PCR-RFLP), we examined the effect of C-1019G on depression symptoms, as evaluated by the Beck Depression Inventory. Data were obtained from 83 volunteers; individuals with depressive symptoms and those with a healthy mood were compared. Results: The results showed that the homozygous C/C genotype was found significantly more frequently in the control group than in individuals with depressive symptoms, particularly among men, and is thus associated with a decreased risk of depressive symptomatology. Conclusions: The C/C genotype could protect against susceptibility to developing depressive symptoms in a rural population in Mexico.

## 1. Introduction

Depression is the most prevalent psychiatric disorder in today’s society, ranking as one of the leading causes of disability globally and significantly contributing to the overall burden of disease worldwide [1]. Its prevalence is higher among populations affected by conflict and has been positively linked to gender and income inequality [2]. In Latin America, the lifetime prevalence of depressive disorder was reported at 12.58% in 2023 [1], while in Mexico, 5.3% of the population has depression [3] and 41.3% present depressive symptoms [4]. Depression is determined by the interaction of multiple environmental factors and genetic variations [1]. The genetic contribution to depression has been demonstrated in genome-wide association studies (GWASs), which have estimated a heritability of 9% captured via single-nucleotide polymorphisms (SNPs) [5,6]. Among the main genes associated with depression and suicidal intent are the encoding proteins involved in the serotonergic system, such as tryptophan hydroxylase (TPH), the serotonin 2A (5-HT2A) receptor, and the serotonin 1A (5-HT1A) receptor [7]. Specifically, 5-HT1A is the most prevalent and widely distributed serotonin (5-HT) receptor in the brain. It acts both as a presynaptic auto-receptor that inhibits the activity of raphe 5-HT neurons and as a postsynaptic receptor involved in mediating the effects of 5-HT across various physiological and emotional processes [8]. In this study, we focused on the C-1019G (rs6295) polymorphism of the 5-HT1A receptor gene (located on chromosome 5q11.2-13). This polymorphism is located in the promoter region and has been associated with substance use disorder, a decreased response to antidepressant use, anxiety, personality disorder, and depression [9,10,11]. Lemonde et al. (2003) [12] reported that the G/G genotype was twice as prevalent in Caucasian individuals with depression and four times more common in those who had died by suicide compared to control subjects. However, the association of the C-1019G (rs6295) polymorphism with depression in rural Mexican populations is unknown. We hypothesized that individuals carrying the G/G genotype of C-1019G may exhibit more depressive symptoms compared to those carrying the C/C and C/G genotypes. Therefore, in this study, we aimed to evaluate the frequency of this polymorphism and its association with depressive symptoms by comparing individuals with healthy and depressive moods in a rural population in Mexico. We found that the C/C genotype might confer protection against susceptibility to developing depressive symptoms in this rural population from Mexico.

## 2. Materials and Methods

### 2.1. Participants

All volunteers were recruited from the psychology department at the Health Center of Sanitary Jurisdiction Number 7, located in the rural municipality of Zapoapan, Ixtaczoquitlán, Veracruz, Mexico, from February to March 2020. Zapoapan is a village located in the central mountainous region of the state of Veracruz. It has a population of 2919 inhabitants, many of whom are bilingual in Nahuatl and Spanish. Three ancient civilizations settled in this area: the Olmec, the Huastec, and the Totonac. As a result, the origins of the current population can be traced to one or more of these three civilizations. Importantly, this population has remained isolated from external cultural and social influences.

In Mexico, the National Institute of Statistics and Geography (INEGI) defines a rural population as people living in localities with fewer than 2500 inhabitants, and an expanded rural population as those residing in areas with fewer than 5000 inhabitants [13]. We included individuals over 18 years of age of both sexes who were Mexican subjects descending from parents and grandparents born in the same community, who donated a blood sample obtained via venipuncture. We excluded individuals who had family relationships outside the local population, those with a history of chronic conditions such as kidney, liver, lung, heart disease, or allergies, as well as pregnant and breastfeeding women. Additionally, we did not exclude individuals with chronic diseases such as diabetes mellitus (DM) and/or arterial hypertension (AH).

### 2.2. Beck Depression Inventory

The Beck Depression Inventory is an effective screening instrument for depression among adults. It contains 21 items and is designed to measure depressive symptoms from the 2 weeks preceding assessment. The score ranges from 0 to 63 points; scores of ≤13, 14–19, 20–28, and 29–63 denote no, mild, moderate, and severe depression, respectively [14]. This inventory was proven to be reliable and valid (Cronbach’s α = 0.80) in a Mexican–American gerontic population [15].

### 2.3. Study Groups

We formed two groups according to the Beck Depression Inventory score. In the control group, we included subjects with a score of ≤13 points (*n* = 51), and in the group with depressive symptoms, we included those with a score of ≥14 points (*n* = 32), indicating depression ranging from mild to severe, according to Segal et al. (2008) [14].

### 2.4. DNA Analysis and Genotyping

**Figure 1:**
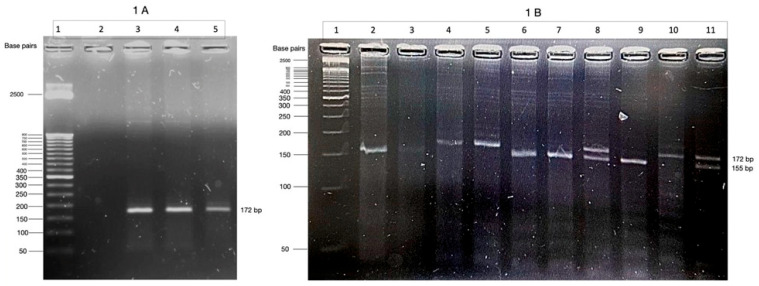
(**A**) PCR products of the 172-base pair (bp) fragment generated after the amplification of the 5-HT1A receptor gene in a 2.0% agarose gel. Lane 1, 50 bp DNA marker; lane 2, negative control; lanes 3–5, polymerase chain reaction (PCR) products obtained from human DNA samples. (**B**) Fragment generated after restriction of enzyme with BseGI enzyme. The digested PCR product had one fragment in 155 bp, corresponding to the C allele, and the undigested PCR product carried the G allele (172 bp). Lane 1, 50 bp DNA marker; lanes 2, 3, 6, 7, and 9 are the homozygous C/C genotype; lanes 4, 5, and 10 are the homozygous G/G genotype; and lanes 8 and 11 are the heterozygous C/G genotype. The images were obtained using an automatic photodocumenter and image analyzer manufactured by BIOBASE.

DNA was isolated from blood leukocytes using the Quick-DNA Kit (Zymo Research, Irvine, CA, USA). The C-1019G (rs6295) polymorphism of the 5-HT1A receptor gene was genotyped using the polymerase chain reaction–restriction fragment length polymorphism (PCR-RFLP) method, based on the methodology used by Rothe et al. (2004) [16] with some modifications. A 172-base pair (bp) fragment was amplified using PCR and the following primers were used, one of which has been modified: F-mod—5′-AGGGAGTAAGGCTGGACTGT-3′ and R—5′-GGAAGAAGACCGAGTGTGTCAT-3′. After amplification, 4 μL of the PCR mixture was visualized using ethidium bromide staining after electrophoresis in a 2.0% agarose gel (Figure 1A). The PCR products were digested with 10 U of BseGI restriction enzyme (New England Biolabs, Ipswich, MA, USA) at 50 °C for 120 min. The products were separated in a 4% agarose gel for 45 min at 100 V. Based on Kato et al. (2009) [17], the digested product had two fragments (17 and 155 bp) corresponding to the C allele, and the undigested PCR product carried the G allele (172 bp) (Figure 1B).

### 2.5. Statistical Analyses

The Shapiro–Wilk test was used to assess the normality of the data, while the Bartlett test was employed to evaluate the homogeneity of variances. The χ^2^ test was conducted to determine whether the data were in Hardy–Weinberg equilibrium. The genotypes were analyzed under the recessive model (G/G + C/G vs. C/C for the C-1019G [rs6295] polymorphism) of the 5-HT1A receptor gene using the chi-square (χ^2^) test and Fisher’s exact test. Logistic regression was applied to evaluate the risk of carrying C-1019G and was adjusted for age and sex. The results are expressed as frequencies or means ± standard deviations for variables with a normal distribution. The Beck Depression Inventory scores did not have a normal distribution, so they are presented as medians and interquartile ranges. A *p* value < 0.05 was considered significant. All analyses were conducted using the statistical program Stata 17.0 (StatCorp LLC, College Station, TX, USA).

## 3. Results

### 3.1. Demographic and Clinical Data of Study Population

#### 3.1.1. Demographic Data

Our study included 83 subjects (19 males and 64 females) aged 59.03 ± 11.91 years, with a body mass index (BMI) of 29.31 ± 6.50 kg/m^2^, systolic blood pressure of 135.50 ± 21.50 mmHg, and diastolic blood pressure of 79.10 ± 13.21 mmHg (Table 1). The case and control groups showed a similar distribution of males and females (*p* = 0.542), and no differences in age, BMI, and blood pressure (*p* > 0.05) were found (Table 1). The average monthly income of the study population was USD 107, which corresponds to a socioeconomic level categorized as poverty according to the INEGI. Additionally, 86.75% of the population had no formal education or had only completed elementary school. Furthermore, 71.08% of the population was engaged in domestic work, 66.27% were married, and 93.98% identified as Catholic.

#### 3.1.2. Clinical Data

According to the medical records, in our population, there were 48 (57.83%) subjects with diabetes mellitus (DM), 20 (24.10%) with arterial hypertension (AH), and 15 (18.07%) with DM plus AH. We observed a higher frequency of patients with both DM plus AH in the group with depressive symptoms (*p* = 0.013) (Table 1).

### 3.2. Depressive Symptoms

**Figure 2:**
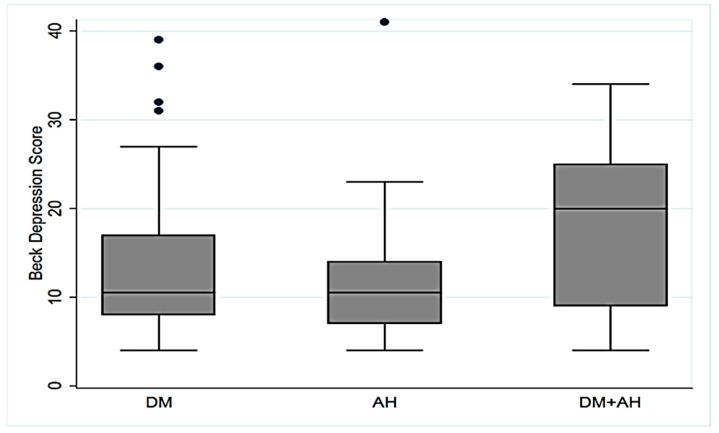
Beck Depression Inventory score by diagnosis for the entire sample. The score was similar between diagnoses (*p* = 0.0794; Kruskal–Wallis test). DM, diabetes mellitus; AH, arterial hypertension. The blue dots are outliers.

Concerning depressive symptoms, the median Beck Depression Inventory score was 11 points (interquartile range [IQR]: 8–18) for the entire sample. The control group had a median score of 8 points (IQR: 6–11), and the group with depressive symptoms had a median score of 21.5 points (IQR: 18–27.5), indicating moderate depression (Table 1). In the population, the Beck Depression Inventory scores did not differ between patients with DM, AH, or DM plus AH (*p* = 0.0794) (Figure 2).

### 3.3. C-1019G (rs6295) Polymorphism of 5-HT1A Receptor Gene

#### 3.3.1. Genotypic Distribution of C-1019G (rs6295) Polymorphism of 5-HT1A Receptor Gene

The genotypic distribution of the C-1019G (rs6295) polymorphism of the 5-HT1A receptor gene was as follows: G/G, 6 (7.23%); C/G, 48 (57.83%); and C/C, 29 (34.94%) (Table 2). The genotypes were in Hardy–Weinberg equilibrium (*p* > 0.05). 

#### 3.3.2. Genotype and Recessive Model Frequencies of C-1019G (rs6295) Polymorphism of 5HT1A Receptor Gene Between Control and Depressive Symptom Groups

We found significant differences in the distribution of the C-1019G (rs6295) polymorphism genotypes between the group with depressive symptoms and the control group; specifically, the frequency of the homozygous C/C genotype was significantly higher in the control group (*p* = 0.035). The same results were also observed for the analysis based on the recessive model (*p* = 0.014) (Table 2). We also performed logistic regression and found that the C/C genotype was associated with a decreased risk of depressive symptoms (*p* = 0.017; odds ratio: 0.28; 95% confidence interval: 0.098–0.79).

#### 3.3.3. Genotype and Recessive Model Frequencies of C-1019G (rs6295) Polymorphism of 5HT1A Receptor Gene Between Control and Depressive Symptom Groups by Sex

Furthermore, a higher frequency of the C/C genotype in males was revealed in the genotypic distribution analysis by sex (*p* = 0.047), as well as the recessive model analysis (*p* = 0.034). The frequencies of the homozygous G/G and heterozygous C/G genotypes were similar in the two groups in all analyses and sub-analyses (by sex) based on genotypic distribution and the recessive model (Table 2).

## 4. Discussion

Due to the lack of studies in Mexico on the relationship between the C-1019G (rs6295) polymorphism of the 5-HT1A receptor gene and symptoms of depression, in this study, we evaluated this association in a rural Mexican population. We found that the proportions of the G/G and C/G genotypes were equal between the control group and the group with depressive symptoms. However, our results showed that the C/C genotype was more frequent in the control group without depressive symptoms, particularly in men, suggesting that being a carrier of the C/C genotype could be a protective factor regarding psychological health in the rural population studied. Our results are consistent with those of Haslacher et al. (2015) [18], who reported that a group of endurance athletes from Austria carrying the C/C genotype had a lower risk of receiving a high depression score. Interestingly, Malaguti et al. (2011) [19] found that patients with depression with the C/C genotype showed an enhanced response to transcranial magnetic stimulation, which is an effective technique in the treatment of this condition, specifically in drug-resistant patients. Additionally, Scutt et al. (2018) [20] found that older people with major depressive disorders carrying the C/C genotype treated with 20 mg of citalopram daily over 4 weeks showed a significant improvement in their geriatric depression score and cognition compared with those carrying other genotypes.

Our results found no association between the G/G genotype and depressive symptoms, since the genotypic frequency was similar across groups; this result contrasts with other reports that found that this genotype is associated with disordered eating in female adolescents [21] and suicide attempts in women [22], and major depression [12], neuroticism traits [23], anxiety [24], panic disorder [25], substance abuse, and psychiatric hospitalization [26] in both women and men. Additionally, the G/G genotype has been associated with major depression and resistance to the antidepressant effects of selective serotonin reuptake inhibitors (SSRIs) and atypical antipsychotics in multiple studies [27]. The above can be explained by the fact that the G/G genotype prevents the repression of the deformed epidermal autoregulatory factor 1 (DEAF1). In turn, DEAF1 represses the 5-HT1A receptor gene [10], resulting in the increased transcription of the 5-HT1A auto-receptor [10,12]. This leads to reduced serotonergic activity, which increases susceptibility to depression and other mental disorders [21,28,29].

Despite the differences in findings regarding the C-1019G (rs6295) polymorphism in our study, some researchers have reported no significant associations between this polymorphism and depressive symptoms. For instance, Zhao et al. (2012) [11] conducted a meta-analysis and found no evidence of an association between this polymorphism and antidepressant responses. Similarly, González-Castro et al. (2013) [30] found no significant association between this polymorphism and suicide attempts in an urban Mexican population from Comalcalco, Tabasco.

In vitro studies suggest that the C-1019G (rs6295) polymorphism could have functional effects on 5-HT1A receptor gene expression, potentially mediated through the altered binding of several transcription factors such as DEAF1/NUDR (deformed epidermal autoregulatory factor 1/nuclear DEAF1-related) and enhancers involved in the regulation of 5-HT transcription [26]. We found that the C/C genotype was associated with a reduced risk of depressive symptomatology. This relationship may be explained by the findings of Donaldson et al. (2016) [26], who demonstrated that DEAF1/NUDR transcription factors act over the C/C genotype, leading to the normal regulation of 5-HT1A receptor gene transcription. Therefore, the C/C genotype regulates 5-HT1A receptor gene expression, leading to adaptive changes in serotonin neurotransmission and neural circuitry involved in mood regulation.

Our results show that all control males carried the C/C genotype. This finding may be explained by a study of adult 5-HT neurons in mice with methyl-CpG binding protein (MeCP2) knockout; this protein interacted with and augmented DEAF1 activity, induced 5-HT1A auto-receptors, and led to a sex-dependent behavioral phenotype: females had reduced anxiety, whereas males showed increased anxiety and reduced depression-like behaviors [31]. Additionally, the acute small interfering RNA (siRNA)-induced knockdown of 5-HT1A auto-receptors in male mice induced a rapid antidepressant-like phenotype within days, and enhanced responses to SSRIs [32].

We found that comorbidities (DM, AH, and DM plus AH) did not influence depressive symptoms. This result contrasts with those of Sandström et al. (2016) [33], who showed that individuals with a diagnosis of hypertension are more commonly diagnosed with depression than those without hypertension. However, our results are consistent with those of Wang et al. (2024) [34], who found no causal relationship between depression and DM.

This study has some limitations. First, these preliminary data suggest the potential role of the C/C genotype as a protective factor against susceptibility to depression, but validation in an independent and larger cohort is essential to confirm this relationship. Second, our results support the theory that certain mutations and polymorphisms may influence receptor responses to neurotransmitters or biologically active substances. However, the pathophysiology of depression is highly complex as well as multifactorial, and thus cannot be fully explained by a single polymorphism. Therefore, different mechanisms have been proposed to describe the development of depression at the biochemical, cellular, anatomical, and physiological levels. These hypotheses are well accepted and involve the monoamine, stress-induced depression, cytokines, neuroinflammation and neuroplasticity, GABA-glutamate-mediated depression, the circadian rhythm of depression, and cholinergic–monoaminergic interaction theory [35]. While each of these hypotheses has its strengths and limitations, none can fully account for all the underlying mechanisms and clinical manifestations of depression.

In conclusion, in our study of a rural Mexican population, the C/C genotype of the C-1019G (rs6295) polymorphism of the 5-HT1A receptor gene could protect against susceptibility to developing depressive symptoms, particularly in men. The results could support physicians in improving the refinement and optimization of antidepressant therapies.

## Figures and Tables

**Table 1:** The demographic and clinical data of the study population.

	All Subjects (*n* = 83)	Control Group (*n* = 51)	Depressive Symptom Group (*n* = 32)	*p*
Age in years (mean ± standard deviation)	59.03 ± 11.91	57.76 ± 11.42	61.06 ± 12.57	0.2214 ^a^
Sex, number (%) Male Female	19 (22.89) 64 (77.11)	12 (23.53) 39 (76.47)	7 (21.875) 25 (78.125)	0.542 ^b^
Body mass index in kg/m^2^ (mean ± standard deviation)	29.31 ± 6.50	29.16 ± 6.13	29.56 ± 7.16	0.8002 ^a^
Blood pressure in mmHg (mean ± standard deviation) Systolic Diastolic	135.50 ± 21.50 79.10 ± 13.21	134.84 ± 21.65 77.9 ± 10.37	136.56 ± 21.56 81.1 ± 16.96	0.7251 ^a^ 0.2974 ^a^
Diagnosis, number (%) Diabetes mellitus Arterial hypertension Diabetes mellitus plus arterial hypertension	48 (57.83) 20 (24.10) 15 (18.07)	32 (62.75) 14 (27.45) 5 (9.80)	16 (50) 6 (18.75) 10 (31.25)	0.252 ^c^ 0.367 ^c^ 0.013 ^c^
Beck Depression Inventory score (median, interquartile range)	11 (8–18)	8 (6–11)	21.5 (18–27.5)	<0.001 ^d^

^a^ Student’s *t*-test; ^b^ Fisher’s exact test; ^c^ chi-square test; and ^d^ Mann–Whitney U test.

**Table 2:** Genotype and recessive model frequencies for the C-1019G (rs6295) polymorphism of the 5-HT1A receptor gene between control and depressive symptom groups.

	**Genotype**				**Recessive Model**		
Entire population *(n* = 83)	G/G	C/G	C/C	*p*	G/G + G/C	C/C	*p*
Controls (*n* = 51)	3	25	23	0.035 ^a^	28	23	0.014 ^b^
Individuals with depressive symptoms (*n* = 32)	3	23	6		26	6	
Females (*n* = 64)							
Controls (*n* = 39)	2	20	17	0.109 ^a^	22	17	0.111 ^b^
Individuals with depressive symptoms (*n* = 25)	0	19	6		19	6	
Males (*n* = 19)							
Controls (*n* = 12)	1	5	6	0.047 ^a^	6	6	0.034 ^a^
Individuals with depressive symptoms (*n* = 7)	3	4	0		7	0	

^a^ Fisher’s exact test; ^b^ chi-square test.

## Data Availability

The data presented in this study are available upon request from the corresponding author, due to the privacy of the participants.

## Abbreviations

The following abbreviations are used in this manuscript:

ADHD	Attention-deficit/hyperactivity disorder
AH	Arterial hypertension
BDI	Beck Depression Inventory
BMI	Body mass index
DEAF-1	Deformed epidermal autoregulatory factor 1
DNA	Deoxyribonucleic acid
dNTPs	Deoxynucleotide triphosphates
DM	Diabetes mellitus
GIRK	G protein-coupled inwardly rectifying potassium channels
GWAS	Genome-wide association
MeCp2	Methyl-CpG binding protein
NUDR	Nuclear DEAF1-related
PCR-RFLP	Polymerase chain reaction–restriction fragment length polymorphism
siRNA	Small interfering RNA
SNP	Single-nucleotide polymorphism
SSRI	Selective serotonin reuptake inhibitor
TPH	Tryptophan hydroxylase
5-HT	Serotonin
5-HT1A	Serotonin 1A
5-HT2A	Serotonin 2A
